# Antidiabetic Potential of *Aronia melanocarpa–*β-Glucan System: From Extraction Optimization Through In Silico Understanding of Activity to Stabilization of Anthocyanins

**DOI:** 10.3390/molecules31132204

**Published:** 2026-06-23

**Authors:** Anna Gościniak, Emmanuelle Lainé, Sandrine Chalancon, Filip Stojceski, Natalia Rosiak, Gabriele Maroni, Judyta Cielecka-Piontek

**Affiliations:** 1 Poznan University of Medical Sciences, Department of Pharmacognosy and Biomaterials, Rokietnicka 3, 60-806 Poznan, Poland; agosciniak@ump.edu.pl (A.G.); nrosiak@ump.edu.pl (N.R.); 2 UMR 454 MEDIS UCA-INRAE, Université Clermont Auvergne, 63000 Clermont-Ferrand, France; emmanuelle.laine@uca.fr (E.L.); sandrine.chalancon@uca.fr (S.C.); 3 SUPSI, IDSIA—Dalle Molle Institute for Artificial Intelligence, Polo Universitario Lugano—Campus Est, Via La Santa 1, 6962 Lugano, Switzerland; filip.stojceski@supsi.ch (F.S.); gabriele.maroni@supsi.ch (G.M.)

**Keywords:** *Aronia melanocarpa*, anthocyanins, β-glucan, stabilization, inhibition of α-amylase

## Abstract

*Aronia melanocarpa* is a rich source of anthocyanins with well-documented antioxidant and antidiabetic potential; however, their application is limited by low stability. In this study, extraction conditions were optimized using response surface methodology, with the highest total polyphenol content obtained at an ethanol concentration of 36.9% (*v*/*v*), an extraction temperature of 34.1 °C, and a solvent-to-solid ratio of 54.5 mL/g. The extract exhibited antioxidant activity and inhibited α-amylase in vitro, with an IC_50_ value of 3.18 ± 0.27 mg/mL, compared with 6.76 ± 0.21 mg/mL for acarbose under the same assay conditions. Molecular modeling suggested that cyanidin derivatives may play a major role in the observed α-amylase inhibitory activity. The optimized extract was subsequently incorporated into yeast-derived β-glucan systems at different ratios to improve anthocyanin stability and formulation performance. Incorporation of β-glucan significantly modified dissolution behavior and reduced anthocyanin degradation in a ratio-dependent manner. The highest stabilization effect was observed for the aronia: β-glucan 1:2 system, in which the degradation rate decreased approximately 4.7-fold.

## 1. Introduction

*Aronia melanocarpa* (chokeberry) is a polyphenol-rich berry, particularly abundant in cyanidin glycosides and phenolic acids such as chlorogenic and neochlorogenic acids [1]. These compounds contribute to its antioxidant, antidiabetic, anti-inflammatory, and cardioprotective properties [2]. Chokeberry extracts have also been reported to inhibit carbohydrate-hydrolyzing enzymes, including α-amylase, supporting their relevance for studies on carbohydrate-digestion modulation [3]. α-Amylase catalyzes the initial hydrolysis of dietary starch into shorter oligosaccharides, which are subsequently converted into glucose by downstream enzymes such as α-glucosidase [4]. For this reason, α-amylase activity represents a relevant early-stage enzymatic marker for evaluating the potential ability of the tested systems to interfere with carbohydrate digestion.

Extraction conditions, including solvent polarity, temperature, and solvent-to-solid ratio strongly influence the composition of chokeberry extracts. Therefore, extraction optimization is required to obtain a reproducible extract for biological testing and formulation development [5]. A chemically characterized extract is necessary for reliable in vitro testing and further formulation development. However, total polyphenol content alone does not fully explain the observed biological effects, as individual compounds may differ in their affinities for biological targets [6]. In this context, molecular docking and molecular dynamics simulations represent complementary exploratory tools that can support the mechanistic interpretation of interactions between selected major polyphenols and the α-amylase binding site [7]. These analyses may help indicate which major aronia constituents could contribute to the observed inhibitory activity; however, they cannot fully explain the activity of the whole extract, which may also depend on minor constituents and additive or synergistic effects.

However, despite their high biological potential, anthocyanins are chemically and physically unstable compounds. They are highly sensitive to temperature, light, and pH, and they readily degrade during processing and storage, as well as in the gastrointestinal tract [8]. Moreover, their relatively low solubility and poor bioavailability further limit their practical application in functional foods and nutraceutical formulations [9]. One promising strategy to overcome these limitations is to incorporate polyphenols into protective carrier matrices.

Yeast-derived β-glucan is a branched β-(1→3)/(1→6) polysaccharide with physicochemical properties distinct from those of cereal β-glucans [10]. Owing to its high degree of branching and strong water-binding capacity, yeast β-glucan readily hydrates and swells in aqueous environments, forming structured networks rather than highly viscous solutions [11]. These properties suggest that yeast β-glucan can form a hydrated polysaccharide matrix that may improve polyphenol dispersion and stability. Its resistance to digestion in the upper gastrointestinal tract may also allow it to reach the colon, where it can interact with beneficial gut microbiota. In addition to its well-established prebiotic and immunomodulatory effects, yeast β-glucan has been investigated as a matrix-forming excipient that may affect the stability and release of incorporated bioactive compounds [12,13,14,15].

The study aimed to provide insight into the antidiabetic mode of action by assessing, using molecular docking, the in silico potential of individual extract constituents to inhibit key carbohydrate-hydrolyzing enzyme, α-amylase. The further aim of this study was to determine whether incorporation of an optimized *Aronia melanocarpa* extract into yeast-derived β-glucan systems can measurably improve anthocyanin stability and dissolution behavior compared with the unformulated extract. The study was designed to determine whether the aronia:β-glucan ratio affects anthocyanin degradation and dissolution behavior under simulated gastrointestinal conditions.

We hypothesized that a chemically characterized *Aronia melanocarpa* extract, obtained under optimized extraction conditions, could be incorporated into a yeast-derived β-glucan matrix to improve anthocyanin stability compared with the unformulated extract. Therefore, extraction optimization was first performed to obtain a reproducible polyphenol-rich extract. The optimized extract was then characterized for α-amylase inhibitory activity, and molecular docking/MD simulations were used to explore the potential contribution of its major constituents to enzyme interaction. Subsequently, the extract was incorporated into β-glucan systems at different ratios to evaluate formulation-dependent dissolution behavior and anthocyanin degradation kinetics.

## 2. Results and Discussion

### 2.1. Optimization of the Extraction Process

The model demonstrated good fitting quality, as indicated by R^2^ = 0.9616 and adjusted R^2^ = 0.9327, showing that the reduced quadratic equation explained most of the variability in TPC. The residual mean square was 1.6379. Importantly, the lack-of-fit was not significant (*p* = 0.156), confirming that the model adequately fitted the experimental data. Therefore, the model was considered suitable for predicting TPC within the investigated range of extraction parameters.

**Figure 1:**
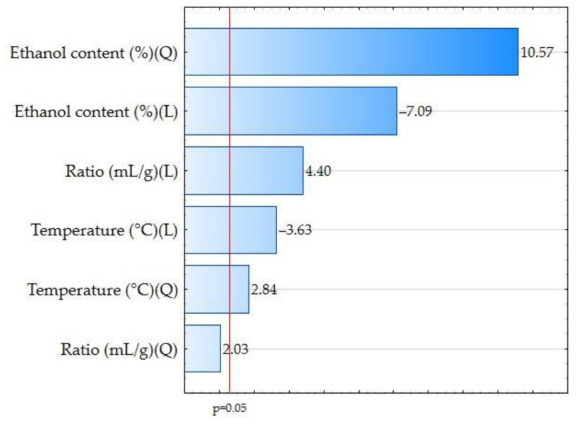
Pareto chart showing the standardized effects of extraction parameters (ethanol concentration, temperature, solvent-to-solid ratio) on the total polyphenol content (TPC).

**Figure 2:**
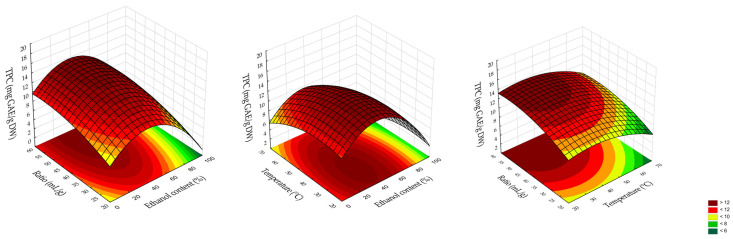
Three-dimensional response surface plots illustrating the combined influence of ethanol concentration and temperature, ethanol concentration and solvent-to-solid ratio, and temperature and solvent-to-solid ratio on the total polyphenol content (TPC) of the aronia extract.

The Pareto chart (Figure 1) indicated that ethanol concentration had the strongest positive impact on total polyphenol content (TPC), followed by the solvent-to-solid ratio, while temperature contributed to a lesser extent. Based on the response surface analysis, the optimal extraction parameters were determined to be an ethanol concentration of 36.9% (*v*/*v*), an extraction temperature of 34.1 °C, and a solvent-to-solid ratio of 54.5 mL/g (Table 1). These conditions corresponded to the highest predicted TPC in the RSM model. The optimized conditions provided a predicted TPC of 14.23 mg GAE/g DW, while the experimentally validated value was 13.85 ± 0.42 mg GAE/g DW, confirming good agreement between the model prediction and the observed response. The moderate ethanol concentration suggests that a hydroalcoholic solvent was more favorable for polyphenol recovery than water or absolute ethanol. The three-dimensional response surface plots visualize the interactive effects of the extraction variables on polyphenol yield (Figure 2).

The selection of a moderate ethanol concentration combined with a relatively low extraction temperature reflects a balance between solvent polarity and the chemical stability of anthocyanins, which are known to be susceptible to thermal and solvent-induced degradation. Recent studies have similarly shown that aqueous ethanol mixtures in the 30–50% range are optimal for extracting anthocyanins and phenolic acids from berry matrices, providing efficient solubilization while limiting structural degradation [16,17,18]. The comparatively lower impact of temperature observed in this study is consistent with reports indicating that increasing extraction temperature may enhance mass transfer but simultaneously accelerate anthocyanin degradation and color loss [19]. Therefore, mild thermal conditions appear particularly advantageous for chokeberry, whose bioactivity is largely driven by thermolabile cyanidin glycosides. The obtained model supports the use of RSM for selecting extraction conditions that maximize TPC in aronia fruit extracts.

### 2.2. Extract Characteristics

#### 2.2.1. HPLC Profile of Active Compounds

**Figure 3:**
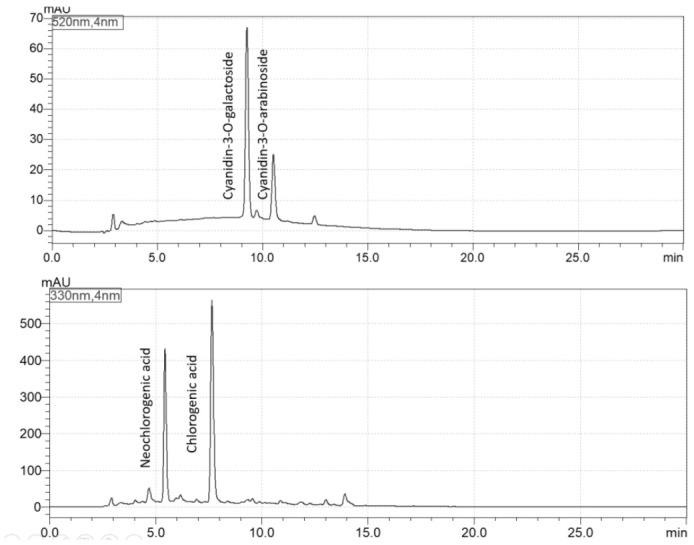
Representative HPLC chromatogram of the optimized *Aronia melanocarpa* extract recorded at 520 nm (anthocyanins) and 330 nm (phenolic acids), showing the major peaks corresponding to cyanidin-3-O-galactoside, cyanidin-3-O-arabinoside, neochlorogenic acid, and chlorogenic acid.

High-performance liquid chromatography (HPLC) analysis revealed the presence of characteristic polyphenolic constituents in the optimized *Aronia melanocarpa* extract. The anthocyanin fraction was dominated by cyanidin-3-O-galactoside and cyanidin-3-O-arabinoside, while phenolic acids such as neochlorogenic acid and chlorogenic acid were also detected (Table 2). The chromatographic profile (Figure 3) showed well-resolved peaks with retention times consistent with those of the reference standards, confirming the identity of the major compounds. Quantitative analysis demonstrated that cyanidin-3-O-galactoside was the most abundant anthocyanin, followed by cyanidin-3-O-arabinoside, which together accounted for the majority of the total anthocyanin content. Neochlorogenic and chlorogenic acids were the main phenolic acids detected in the extract.

The HPLC analysis of the optimized *Aronia melanocarpa* extract revealed a polyphenolic profile characteristic of chokeberry fruits, dominated by cyanidin-based anthocyanins and chlorogenic acid derivatives. The predominance of cyanidin-3-O-galactoside and cyanidin-3-O-arabinoside is consistent with previous phytochemical studies, which identify these compounds as the principal anthocyanins in aronia irrespective of cultivar or origin [20]. Reports by Sasmaz et al. [21] and Dobros et al. [22] describe the anthocyanin content in chokeberries, noting that it varies by variety and stage of development. In addition, the presence of neochlorogenic and chlorogenic acids is consistent with the literature, which indicates that these phenolic acids constitute a major fraction of aronia polyphenols and may contribute to its antioxidant and metabolic activity, as reported by Zielińska et al. [23]. The relatively lower concentrations of cyanidin glycosides compared with chlorogenic and neochlorogenic acids may reflect differences in extraction selectivity and compound stability. Previous HPLC-DAD studies have shown that *Aronia melanocarpa* contains both anthocyanins and significant amounts of hydroxycinnamic acids, including chlorogenic and neochlorogenic acids, and that their relative levels vary depending on harvest time, maturity stage, cultivation region, and processing conditions [23].

#### 2.2.2. In Vitro Activity Results

The lyophilized *Aronia melanocarpa* extract exhibited inhibitory activity against α-amylase, a key enzyme involved in carbohydrate digestion. The extract demonstrated an IC_50_ of 3.18 ± 0.27 mg/mL, approximately twofold lower than that of the reference drug acarbose (Table 3), showing a lower IC_50_ value than acarbose under the applied assay conditions.

The chokeberry extract showed substantial antioxidant activity in both ABTS and CUPRAC assays, although it was less potent than ascorbic acid, as expected for a complex polyphenol mixture (Table 4). In the ABTS assay, ascorbic acid was about 21-fold more active, reflecting its strong radical-scavenging ability as a small, pure molecule. In contrast, in the CUPRAC assay, the difference was reduced to about six-fold, indicating a relatively high reducing power of chokeberry polyphenols. This suggests that the antioxidant activity of the extract is primarily driven by electron-transfer mechanisms characteristic of anthocyanins and chlorogenic acids [24]. Such a redox profile is particularly relevant for limiting oxidative stress associated with diabetic pathology [25].

The α-amylase inhibitory activity observed for the Aronia melanocarpa extract is consistent with reports showing that berry polyphenols can interact with carbohydrate-hydrolyzing enzymes [26,27]. The reported inhibitory activity of West Mexico Berries showed that berry-derived extracts inhibited α-amylase, with IC_50_ values ranging from 4.02 to 7.66 mg/mL [28]. Antioxidant activity is relevant in antidiabetic strategies because oxidative stress contributes to insulin resistance and β-cell dysfunction. Polyphenolic antioxidants can mitigate these processes while simultaneously modulating carbohydrate-digesting enzymes, thereby supporting glucose homeostasis [29]. Berry anthocyanins have been shown to reduce oxidative stress and modulate glucose metabolism in vitro, suggesting that their antioxidant and antidiabetic effects may act in a complementary manner [30]. Results demonstrate that the antidiabetic and antioxidant activities of the optimized aronia extract are consistent with contemporary evidence on berry polyphenols. However, because the specific contributions of individual polyphenolic constituents to α-amylase inhibition remain unclear, identifying the key bioactive compounds is crucial. To further examine which major constituents could contribute to this activity, the four dominant compounds identified in the extract were selected for molecular modeling.

#### 2.2.3. In Silico Results

**Figure 4:**
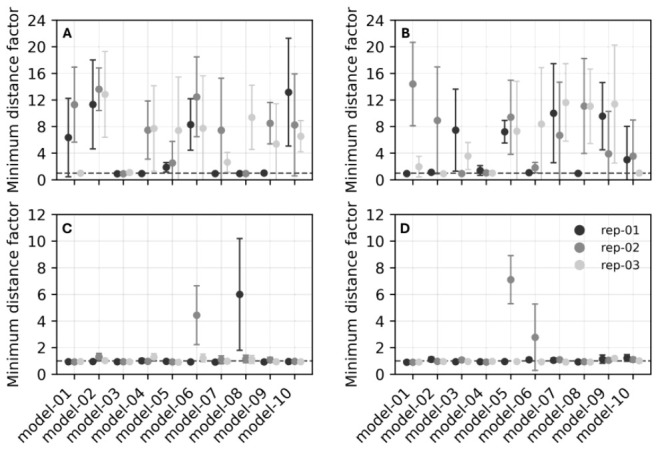
Minimum distance factor analysis between the pancreatic α-amylase (HPA) binding pocket residues and (**A**) chlorogenic acid (CGA), (**B**) neochlorogenic acid (NCGA), (**C**) cyanidin-3-O-galactoside (C3G), and (**D**) cyanidin-3-O-arabinoside (C3A). The analysis was performed by calculating the mean and standard deviation of the minimum distance between each ligand and the HPA binding site, normalized by a factor of 0.175. Average values below 1 (dashed line) indicate that the ligand remains within the cut-off distance throughout the simulation. The analysis includes all replicas (rep 1–3) for all docking models (models 1–10).

The molecular docking was used to obtain 10 binding poses for each ligand that were used as starting points for MD simulations in 3 replicas. Following molecular docking, 10 binding poses for each ligand were subjected to MD simulations in 3 replicas. The stability and persistence of ligand-protein interactions were first evaluated using a minimum distance factor analysis, which provides a quantitative descriptor of a ligand’s ability to remain within a strong interaction distance of the HPA binding site during simulations (Figure 4). This analysis computes the minimum ligand-protein distances observed during the MD simulations, normalized by 0.175. The results are shown as average and standard deviation. A minimum distance factor close to or below 1 indicates that the ligand can maintain close contacts with the binding site residues, while values larger than 1 are indicative of loose interactions. Values greater than 3 indicate the absence of interactions with residues in the HPA binding site.

In detail, CGA and NCGA systematically display minimum distance factor values well above 1 across the different docked models and replicas (Figure 4A,B). This behavior suggests that these ligands are unable to remain stably within a strong interaction distance of the HPA binding pocket. In contrast, C3G and C3A exhibit a markedly different interaction pattern (Figure 4C,D). For these ligands, most docked models and replicas show minimum distance factor values close to 1 or even below 1, indicating persistent interactions with the HPA binding site. This result showed a clear preference for HPA toward anthocyanin-based ligands, with C3G and C3A showing significantly greater binding persistence than CGA and NCGA.

A detailed view of the minimum distance analysis for all replicas of all docked models is reported in Figures S1–S4 of the Supporting Information. In addition, the corresponding averages and standard deviations for each system are provided in Tables S1–S4.

**Figure 5:**
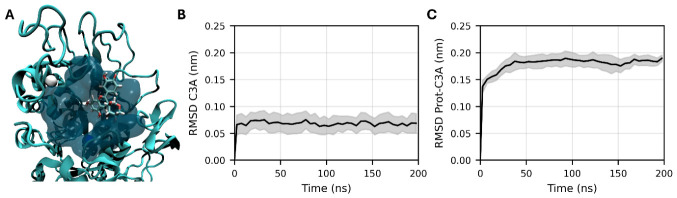
(**A**) Qualitative representation of the pancreatic α-amylase (HPA) binding site (dark blue surface) with the ligand bound within the cavity. (**B**) Root-mean-square deviation analysis (RMSD) of the cyanidin-3-O-arabinoside (C3A) ligand and (**C**) RMSD analysis of the protein-ligand complex (HPA-C3A). Both RMSD analyses are presented as the average (black line) with the corresponding standard deviation (grey shaded area), calculated using 5 ns time windows.

Based on the minimum distance factor analysis, systems exhibiting values below 1 were selected for further investigation. Among these, one representative model was chosen for in-depth analysis by jointly considering the minimum distance factor (the lowest value across all 3 replicas) and the root mean square deviation (RMSD) behavior. Figure 5B,C show the RMSD over the 200 ns of the ligand and of the protein-ligand complex of the model-01 of the HPA-C3A system. These analyses are calculated using 5 ns time windows and reported as the average with the corresponding standard deviation.

In both cases, the RMSD reaches a plateau early in the simulation and remains stable throughout the entire trajectory, indicating the absence of large conformational rearrangements and confirming the overall stability of the complex. The RMSD profiles of all the other systems characterized by minimum distance factors below 1 are reported in Figures S5–S8 of the Supporting Information.

**Figure 6:**
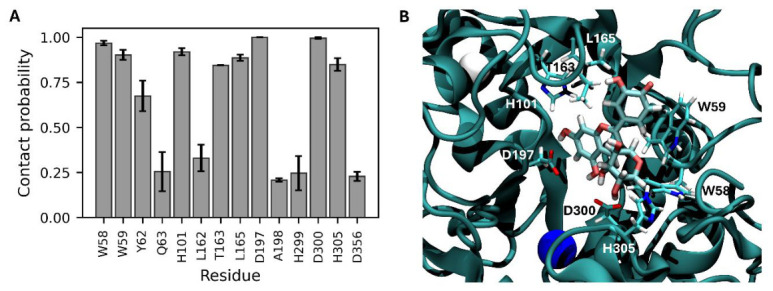
(**A**) Contact probability analysis of the pancreatic α-amylase (HPA) residues with cyanidin-3-O-arabinoside (C3A) ligand. A cut-off distance of 0.28 nm was used. (**B**) Qualitative representation of HPA residues interacting with C3A. Only residues with a contact probability greater than 0.75 are shown.

To identify residues most frequently involved in C3A binding, contact probability analysis was performed on model-01 of the HPA-C3A system. The analysis was carried out using a distance cutoff of 0.28 nm, and the results are presented as a bar plot showing the average contact probability and its associated standard deviation across the three independent replicas (Figure 6A).

The CP analysis reveals that C3A establishes contacts with several residues lining the HPA binding site. In particular, the following residues were identified as being involved in the interaction: W58, W59, Y62, Q63, H101, L162, T163, L165, D197, A198, H299, D300, H305 and D356. Among these, a subset of residues shows high contact probabilities (CP > 0.75), indicating a dominant role in stabilizing the ligand within the binding pocket. These key residues are W58, W59, H101, T163, L165, D197, D300 and H305. A qualitative structural representation of these residues and their spatial relationship with C3A is shown in Figure 6B, highlighting the dense interaction network formed between the ligand and the catalytic region of HPA.

The in silico analysis revealed differences in binding behavior among the investigated polyphenolic constituents. Anthocyanin derivatives exhibited interactions within the α-amylase binding site, as confirmed by low minimum distance factors and stable RMSD profiles, whereas chlorogenic acid derivatives failed to maintain close contact with the enzyme. This observation is consistent with previous studies reporting that glycosylated anthocyanins can effectively interact with carbohydrate-hydrolyzing enzymes through hydrogen bonding and stabilization within the active site [31]. Molecular docking analyses have demonstrated that anthocyanins can bind α-amylase, often forming interactions with catalytic residues and contributing to competitive inhibition. Furthermore, structure–activity relationship studies indicate that cyanidin-based glycosides exhibit stronger inhibitory activity than other phenolic compounds, highlighting the importance of molecular structure and glycosylation patterns in enzyme binding [31].

Similar trends have been reported in computational and experimental studies, showing that anthocyanins and anthocyanidins can act as effective inhibitors of carbohydrate-hydrolyzing enzymes, with their activity largely determined by their ability to form stable hydrogen bonds and π–π interactions within the enzyme active site [32].

### 2.3. FT-IR Spectroscopy of Aronia melanocarpa- β-Glucan System

**Figure 7:**
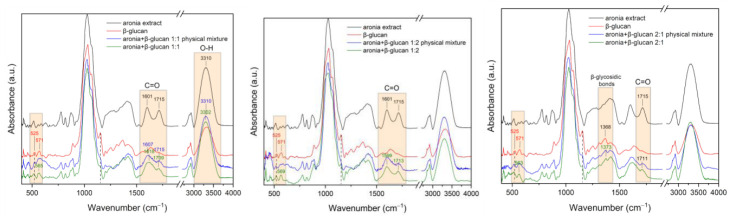
ATR-FTIR spectra of lyophilized *Aronia melanocarpa* extract, β-glucan, and aronia–β-glucan systems (1:1, 2:1, and 1:2, *w*/*w*).

Lyophilized *Aronia melanocarpa* extract was mixed with yeast-derived β-glucan in water at extract-to-carrier ratios of 1:1, 2:1, and 1:2, and the obtained dispersions were freeze-dried. The resulting systems were then analyzed by ATR-FTIR to evaluate possible interactions between aronia polyphenols and the β-glucan matrix. Fourier transform infrared (FT-IR) spectroscopy was applied to evaluate potential intermolecular interactions between *Aronia melanocarpa* extract and β-glucan in the developed systems (Figure 7). The FT-IR spectrum of the aronia extract was characterized by a broad O–H stretching band in the 3200–3600 cm^−1^ region, bands associated with aromatic C=C and conjugated C=O stretching vibrations in the 1500–1700 cm^−1^ range, and intense C–O stretching bands between 1000 and 1200 cm^−1^, typical of glycosylated polyphenols [33,34,35]. β-Glucan exhibited a dominant broad O–H stretching band around 3300–3400 cm^−1^ and strong C–O–C and C–O stretching vibrations of glucopyranose units in the 1000–1150 cm^−1^ region [36].

In the physical mixtures of aronia extract and β-glucan, the recorded spectra largely resembled a superposition of the individual components, with β-glucan bands dominating due to its greater mass contribution. No substantial shifts in characteristic bands were observed, indicating limited molecular interaction in the physical mixtures. In contrast, the lyophilized aronia–β-glucan systems exhibited subtle but reproducible spectral changes. The observed broadening and slight shifts of bands associated with hydroxyl and carbonyl groups may indicate changes in the local hydrogen-bonding environment. These spectral changes are consistent with possible non-covalent interactions between aronia polyphenols and β-glucan, mainly through hydrogen bonding; however, FT-IR provides indirect evidence and does not allow definitive confirmation of specific molecular interactions. These effects were most pronounced in the β-glucan-rich system (1:2), while less evident in the aronia-rich formulation (2:1), indicating a composition-dependent interaction pattern.

Additionally, minor shifts and changes in intensity were detected in the 1600–1700 cm^−1^ region, attributed to carbonyl and aromatic vibrations of aronia polyphenols. These changes were more evident in the lyophilized systems than in the corresponding physical mixtures, suggesting closer molecular contact between the polyphenolic constituents and the polysaccharide matrix. Overall, the FT-IR spectra support the presence of weak non-covalent interactions, mainly hydrogen bonding, between aronia polyphenols and β-glucan.

### 2.4. Dissolution Profiles

**Figure 8:**
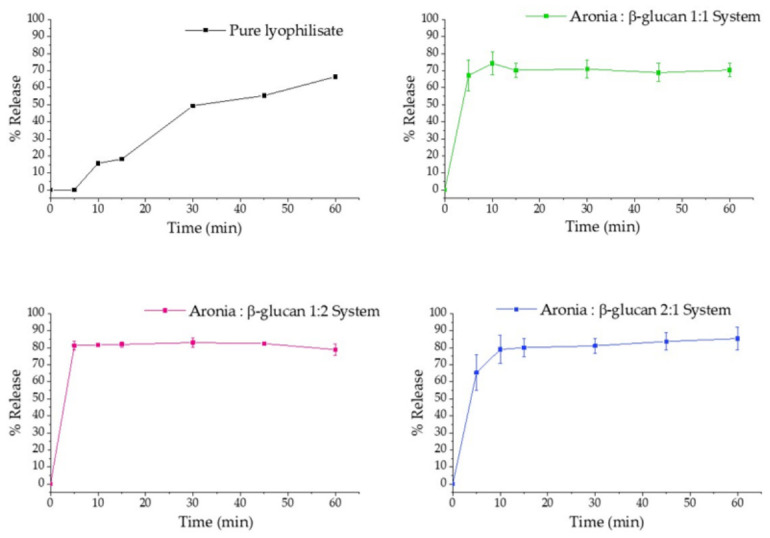
Dissolution profiles of marker compound (chlorogenic acid) from freeze-dried chokeberry formulations containing different ratios of β-glucan (1:1, 1:2, 2:1 *w*/*w*) and from the control lyophilisate without β-glucan, obtained using the USP IV flow-through cell in simulated gastric fluid (pH 1.2).

**Figure 9:**
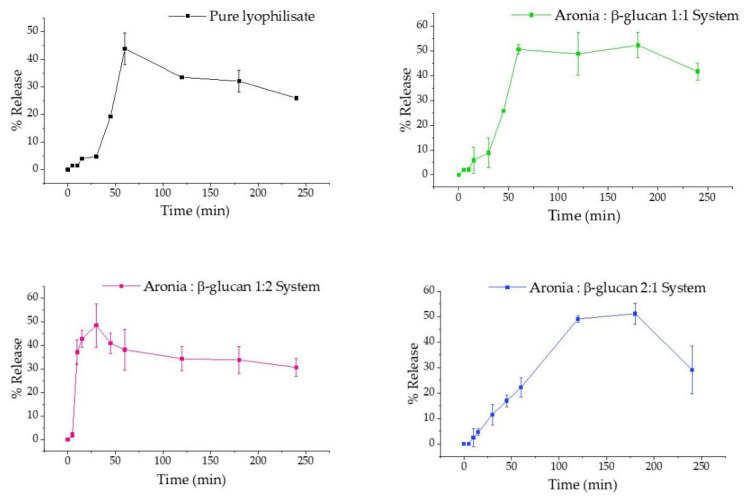
Dissolution profiles of marker compound (chlorogenic acid) from freeze-dried chokeberry formulations containing different ratios of β-glucan (1:1, 1:2, 2:1 *w*/*w*) and from the control lyophilisate without β-glucan, obtained using the USP IV flow-through cell in intestinal fluid (pH 6.8) at 37 °C.

Formulations containing β-glucan modified dissolution profiles compared with the pure chokeberry lyophilisate in both SGF (pH 1.2) and SIF (pH 6.8), with a pronounced dependence on the aronia:β-glucan ratio (Figure 8 and Figure 9). Chlorogenic acid was used as a marker compound to compare the release behavior of different aronia–β-glucan systems. Its selection was based on its abundance in the extract and reliable quantification during the entire dissolution experiment. The dissolution experiments were performed using a closed-loop USP Apparatus 4 configuration under non-sink conditions. This experimental setup was intentionally selected to compare the influence of the β-glucan matrix on release behavior under dynamic recirculating conditions and fixed medium volume. In polyphenol–polysaccharide systems, apparent release may depend not only on dissolution of the marker compound, but also on matrix hydration, swelling, dispersion, and reversible interactions between phenolic compounds and the hydrated polysaccharide network. Therefore, the closed-loop non-sink setup was considered useful for evaluating formulation-dependent behavior of the aronia–β-glucan systems under identical experimental conditions. Although such conditions do not fully reproduce the physiological gastrointestinal environment, they provide useful information regarding formulation-dependent release characteristics. Therefore, the observed differences between formulations should be interpreted primarily as comparative performance indicators rather than direct predictors of in vivo gastrointestinal release.

In our system, β-glucan-containing formulations achieved higher chlorogenic acid levels than the pure lyophilisate, and the magnitude of this effect increased with the proportion of β-glucan, indicating that β-glucan influenced the dissolution behavior of the system. Literature data show that β-glucan can modulate polyphenol availability in different directions depending on the experimental model and readout: in an in vitro gastrointestinal digestion model. Jakobek et al. [13] reported that β-glucan entrapped aronia phenolics and lowered the quantities of recovered phenolic acids and anthocyanins, and additionally demonstrated adsorption of aronia phenolics onto β-glucan at pH 1.5, 3.0 and 7.0, with a clear dependence on β-glucan concentration. Previous studies using cereal β-glucans have shown reduced phenolic recovery during simulated digestion, which was attributed to interactions between phenolic compounds and the polysaccharide matrix [14]. However, cereal β-(1→3)/(1→4)-glucans differ structurally and physicochemically from yeast-derived β-(1→3)/(1→6)-glucans. In the present study, the yeast β-glucan matrix promoted apparent chlorogenic acid release under dynamic recirculating conditions, which may be related to its hydration, swelling, and matrix-disintegration properties rather than to a simple adsorption-driven mechanism.

β-Glucan hydrates rapidly and swells in aqueous media, thereby improving the wetting of solid matrices and facilitating their disintegration. In the present study, a yeast-derived β-glucan with a β-(1→3)/(1→6) linkage pattern was utilized, characterized by a branched structure and high hydration capacity. The physicochemical properties of β-glucans strongly depend on their source and linkage pattern, with branched yeast β-(1→3)/(1→6)-glucans exhibiting higher swelling and hydration capacity, whereas cereal β-(1→3)/(1→4)-glucans primarily increase solution viscosity [10,37]. Under the applied closed-loop conditions, these hydration and swelling properties likely played a dominant role by enhancing solvent penetration and promoting continuous re-dispersion of released compounds within the circulating medium. Therefore, the observed enhancement of dissolution should be interpreted as a combined effect of matrix-driven disintegration and system hydrodynamics rather than solely as increased intrinsic solubility of polyphenols. Chlorogenic acid should be interpreted as an analytical marker of the phenolic acid fraction rather than as a direct surrogate for anthocyanin release. Anthocyanins and chlorogenic acid may differ in release behavior due to differences in chemical stability, solubility and interactions with the carrier matrix. The closed-loop non-sink configuration may be considered advantageous for comparative assessment of such polyphenol–polysaccharide systems, because it preserves the released marker within the same experimental environment and therefore allows matrix-dependent phenomena, including hydration, swelling, dispersion, retention, and possible reversible re-association, to contribute to the apparent release profile. These effects could be partly masked under strict sink conditions, where the released compound is continuously diluted or removed from the dissolution environment. Nevertheless, the lack of sink-condition validation should be regarded as a limitation when extrapolating these comparative dissolution profiles to other release conditions or to in vivo gastrointestinal behavior.

### 2.5. Anthocyanin Degradation Kinetics in Aronia melanocarpa- β-Glucan System

The degradation kinetics of anthocyanins in the lyophilized aronia extract and aronia–β-glucan systems were evaluated at 85 °C. The accelerated condition at 85 °C was applied as a thermal stress model to compare the relative protective effect of β-glucan against anthocyanin degradation within a short experimental time. This condition was not intended to directly reproduce conventional storage conditions, but to provide a controlled comparative assessment of formulation performance under intensified thermal stress. Such elevated-temperature testing may be relevant for assessing formulation robustness during thermal exposure and for comparing the relative stabilizing effect of β-glucan under intensified stress conditions. However, it should not be interpreted as evidence of protection during gastrointestinal transit. Long-term storage stability and gastrointestinal protection would require additional studies under milder and physiologically relevant conditions, including 37 °C and different pH environments.

**Figure 10:**
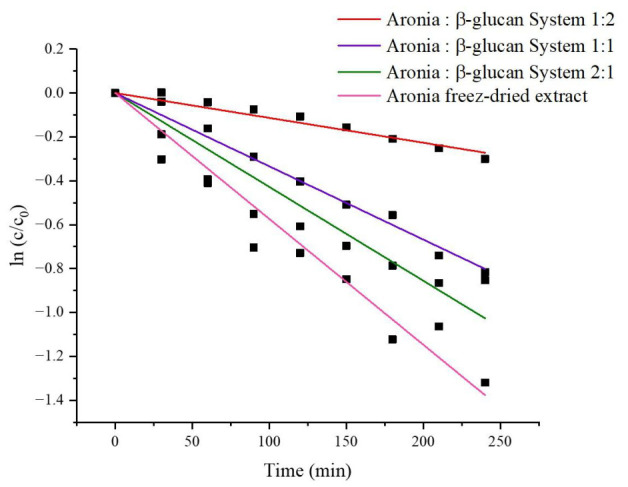
Thermal degradation profiles of anthocyanins in lyophilized aronia extract and aronia–β-glucan systems at 85 °C, illustrating the protective effect of β-glucan on anthocyanin stability.

The unmodified extract showed the fastest degradation (k = 9.56 × 10^−5^ s^−1^, t_1/2_ ≈ 2.0 h). Incorporation of β-glucan significantly slowed anthocyanin decomposition, especially in the 1:2 system, where k decreased to 2.05 × 10^−5^ s^−1^ and t_1/2_ increased fivefold to ~10.7 h. These results confirm the protective effect of β-glucan, which is likely related to the hydrogen bonding and entrapment of polyphenols within the polysaccharide matrix. The numerical kinetic parameters are summarized in Table 5, and the corresponding degradation profiles are presented in Figure 10.

The observed stabilization is consistent with previous reports showing that carbohydrate-based matrices can improve anthocyanin stability by limiting molecular mobility, reducing exposure to degrading factors, and promoting non-covalent interactions [38]. Although β-glucan has been less frequently investigated in the context of anthocyanin degradation kinetics, its physicochemical properties closely align with the stabilization mechanisms identified in these studies [39,40]. As a polysaccharide capable of strong hydration, swelling, and network formation, β-glucan can reduce anthocyanin mobility, limit exposure to degrading factors, and engage in non-covalent interactions such as hydrogen bonding [41]. Thus, although β-glucan has been less studied as a carrier for anthocyanins, the observed stabilization is consistent with known mechanisms of polysaccharide-based protection.

## 3. Materials and Methods

### 3.1. Aronia Extract Optimization

The optimization of aronia fruit extraction was designed to enhance the yield of bioactive compounds, particularly polyphenols. A Box–Behnken experimental design was employed to systematically evaluate the influence of three critical parameters: ethanol concentration in the solvent (% *v*/*v*), extraction temperature (°C), and the solvent-to-solid ratio (mL/g) (Table 6). The selected ranges for these variables were based on preliminary trials and literature data. During the preliminary stage, ethanol concentration, extraction temperature, solvent-to-solid ratio, extraction time, and acid concentration in the solvent were considered. Ethanol concentration, temperature, and solvent-to-solid ratio were selected as independent variables because they were expected to have the strongest influence on polyphenol recovery. Other parameters, including extraction time and formic acid concentration, were kept constant to reduce experimental variability and maintain comparable extraction conditions. Based on these observations, ethanol concentration (0–100%, *v*/*v*), extraction temperature (20–70 °C), and solvent-to-solid ratio (20–60 mL/g) were selected as independent variables in the Box–Behnken design, whereas extraction time was kept constant at 60 min. The model adequacy was confirmed based on ANOVA, determination coefficients, residual mean square, and the non-significant lack-of-fit test. For each experimental condition, 500 mg of dried aronia berries were extracted with solutions containing 0.1% formic acid. The extractions were carried out in an ultrasonic bath at the designated temperature for 60 min. The filtrates were brought to a final volume and stored at 4 °C. The total polyphenol content (TPC) was determined using the Folin–Ciocalteu method and expressed as mg gallic acid equivalents (GAE) per gram of dry weight. The data were subjected to response surface methodology (RSM) analysis to determine the optimal extraction conditions.

### 3.2. Lyophilisation

The optimized aronia extract was freeze-dried using a LyoQuest −85 lyophilizer (Telstar, Terrassa, Spain) at −85 °C and 0.2 mbar for 96 h. After lyophilization, the dry mass was ground into a fine powder and stored at 4 °C in sealed containers.

### 3.3. HPLC Analysis

The assessment of active compound content was conducted using a mobile phase consisting of 5% formic acid in water (A) and 5% formic acid in methanol (B). A column was Reprosphere C18 column 250 mm × 4.6 mm, 5 µm(Dr. Maisch GmbH, Ammerbuch-Entringen, Germany) with a flow rate of 1 mL/min and a column temperature of 30 °C. The detection of active compounds was performed at 520 nm and 330 nm. The injection volume was 10 µL. The gradient was as follows: 0–15 min (B = 15–55%), 15–20 min (B = 55–65%), 20–25 min (B = 65–70%), 25–27 min (B = 70–75%), and 27–30 min (B = 75–15%). The validation parameters of the HPLC method, including linearity, precision, LOD, and LOQ, are provided in Table S5 in the Supplementary Materials.

### 3.4. In Vitro Antidiabetic Activity

The inhibitory activity of the aronia extract against α-amylase was determined according to the method described by Gościniak et al. [14]. Porcine pancreatic α-amylase was used as a standard in vitro model enzyme commonly applied for screening the α-amylase inhibitory activity of plant extracts and natural products. The extract was incubated with porcine pancreatic α-amylase and a starch solution at 37 °C. The reaction was stopped with DNSA reagent, then heated. Absorbance was measured at 540 nm. Acarbose was used as a positive control, and percentage inhibition was calculated.

### 3.5. In Silico Antidiabetic Activity

Human pancreatic α-amylase (HPA) was selected as the target enzyme for the in silico investigation of the antidiabetic potential of the four ligands considered in this study (CGA, NCGA, C3G, and C3A). This choice was motivated by both biological and computational considerations. From a biological perspective, HPA is a well-established therapeutic target for modulating postprandial glucose levels, and a large number of experimentally determined structures are available in the Protein Data Bank, providing reliable structural information for modeling studies From a computational perspective, the availability of a high-resolution HPA crystal structure (PDB ID: 6OCN) provided a reliable structural basis for molecular docking and molecular dynamics simulations. 6OCN is currently among the highest-resolution crystallographic structures available for pancreatic α-amylase (1.15 Å resolution) [42]. The exceptional resolution of this structure significantly increases the reliability of atomic coordinates, side-chain orientations, hydrogen-bonding geometries, and binding-pocket definition, all of which are critical parameters in molecular docking and MD simulations [43,44].

Although the in vitro inhibition assay was performed using porcine pancreatic α-amylase, the in silico analysis employed human pancreatic α-amylase because of the availability of a high-resolution crystallographic structure. Human and porcine pancreatic α-amylases show high sequence similarity and conservation of the catalytic and binding-site residues, including W58, W59, H101, L165, D197, D300, and H305. Therefore, the HPA model was considered suitable for qualitative mechanistic interpretation, but not for direct quantitative reproduction of the experimental IC_50_ values.

The atomistic structure of human pancreatic α-amylase (HPA) was obtained from X-ray diffraction data (PDB ID: 6OCN [42]). Four small molecules were investigated in this study: cyanidin-3-O-galactoside (C3G), cyanidin-3-O-arabinoside (C3A), chlorogenic acid (CGA) and neochlorogenic acid (NCGA). Partial atomic charges for all compounds were derived using the abcg2 charge method [45]. Chlorogenic acid and neochlorogenic acid carried a total charge of −1, while cyanidin-3-O-galactoside and cyanidin-3-O-arabinoside were modeled as neutral molecules. Ligand topologies were generated using the ACPYPE [46] tool in combination with the General Amber Force Field 2 (GAFF2) [47], following protocols previously applied in the literature [48,49].

Protein–small molecule docking was performed using the HADDOCK 2.4 webserver [21], which implements a data-driven docking approach by integrating prior structural information into an energy-based protocol that combines rigid-body docking, semi-flexible refinement, and explicit solvent refinement. For all compounds, the binding pocket of HPA was defined according to the interaction site reported in the literature for the 6OCN structure [42]. For each ligand, the first 10 docking poses generated by HADDOCK were selected as starting configurations for subsequent molecular dynamics (MD) simulations.

Production molecular dynamics simulations were performed for 200 ns per replica in three independent replicas for each selected docking pose. Trajectory analyses included minimum distance factor analysis, RMSD profiles, and contact probability analysis. Detailed simulation parameters, including system preparation, solvation, ion conditions, equilibration, force-field settings, constraint settings, and trajectory-analysis protocols, are provided in the Supplementary Materials Section S1 [50,51,52,53,54,55,56,57,58].

### 3.6. Preparation of Aronia–β-Glucan Systems

Lyophilized aronia extract was mixed with β-glucan in ratios 1:1, 1:2, and 2:1 (*w*/*w*) in water containing 0.1% formic acid. The mixtures were stirred for 24 h at room temperature under a cover, then lyophilized to obtain the dry systems.

### 3.7. FT-IR Spectroscopy

Fourier-transform infrared (FTIR) spectroscopy was used to evaluate potential interactions between aronia extract and β-glucan in the prepared systems. Spectra were recorded using an ATR-FTIR spectrometer (Shimadzu, Kyoto, Japan) over 4000–400 cm^−1^ with a resolution of 4 cm^−1^. The spectra of individual components (aronia extract and β-glucan) and the lyophilized systems (1:1, 1:2, and 2:1) were compared. Characteristic peaks associated with hydroxyl groups (–OH), aromatic rings, and polysaccharide structures were analyzed to detect possible hydrogen bonding or other interactions.

### 3.8. Dissolution Testing

Dissolution testing was performed using USP Apparatus 4 (CE 7 Smart with CP7 piston pump, Sotax AG, Aesch, Switzerland) in a closed-loop flow-through configuration. The system operated in a closed-loop configuration, allowing continuous recirculation of the dissolution medium throughout the experiment. This configuration was selected as a controlled comparative model to evaluate the release behavior of the different aronia–β-glucan systems under reproducible dynamic flow conditions. The approach enabled direct comparison of formulation performance and assessment of how the β-glucan matrix influenced the release of phenolic compounds. Chlorogenic acid was selected as the analytical marker for dissolution testing because it was one of the major phenolic constituents of the aronia extract and could be reliably quantified under the applied HPLC conditions throughout the release experiment.

Approximately 0.75 g of each lyophilized formulation was placed in the flow-through cell, and the dissolution medium was pumped through the system at a constant flow rate of 20 mL/min. The experiments were conducted at a controlled temperature (37 ± 0.5 °C) to simulate physiological gastrointestinal conditions. The system operated in a closed-loop configuration, allowing the dissolution medium to be recirculated throughout the experiment. This configuration enables evaluation of release under non-sink and recirculating conditions, which may better reflect the behavior of polyphenol-rich systems. Dissolution experiments were carried out separately in two media (250 mL of dissolution medium): simulated gastric fluid (SGF, pH 1.2) and simulated intestinal fluid (SIF, pH 6.8).

Samples (3 mL each) were collected at the following time points: 5, 10, 15, 30, and 45 min; 1, 2, 3, 4, 5, and 6 h. The withdrawn samples were not replaced, and the cumulative withdrawn volume (33 mL) was considered in the calculation of release profiles. The collected samples were filtered through a 0.22 μm nylon membrane filter prior to analysis. Chlorogenic acid was selected as a representative phenolic marker for dissolution testing because it was one of the major phenolic constituents of the aronia extract and could be reliably quantified throughout the release experiment. The dissolution study was designed to compare the release behavior of the different aronia–β-glucan systems.

### 3.9. Degradation Kinetics

The stability of aronia extract in lyophilized aronia–β-glucan systems was evaluated under accelerated thermal conditions to assess the protective capacity of the matrix. Samples were incubated at 85 °C for 4 h. The total anthocyanin content was quantified using high-performance liquid chromatography (HPLC), based on the sum of cyanidin-3-galactoside and cyanidin-3-arabinoside, which were identified as the dominant anthocyanins in aronia. Degradation kinetics were modeled using a first-order reaction equation. The degradation rate constant (k), correlation coefficient (r), and half-life (t_0.5_) were calculated to enable quantitative comparison of anthocyanin stability between the different formulation ratios.

### 3.10. Statistical Analysis

For statistical comparison of results between the tested groups, an unpaired Student’s *t*-test was used for comparisons between two groups, whereas one-way analysis of variance (ANOVA) followed by Tukey’s post hoc test was applied for comparisons involving more than two groups. Differences were considered statistically significant at *p* < 0.05.

## 4. Conclusions

This study showed that yeast-derived β-glucan can improve the stability of anthocyanins from *Aronia melanocarpa* extract in a ratio-dependent manner. Among the tested systems, the aronia:β-glucan 1:2 formulation provided the strongest protective effect, reducing the anthocyanin degradation and increasing the half-life under accelerated thermal conditions. This suggests that increasing the β-glucan fraction improved the protection of anthocyanins under thermal stress.

FT-IR analysis indicated the presence of non-covalent interactions, primarily hydrogen bonding, between aronia polyphenols and β-glucan. In dissolution studies, β-glucan-containing systems also modified the release profile of phenolic compounds compared with the unformulated lyophilisate, indicating that the matrix influenced both anthocyanin protection and formulation behavior. The optimized extract retained antioxidant activity and inhibited α-amylase in vitro, while molecular modeling suggested that cyanidin glycosides may contribute to this effect through more stable interactions with the enzyme-binding site than chlorogenic acid derivatives.

These results support the potential of yeast-derived β-glucan as a functional carrier for anthocyanin-rich aronia extracts. Among the tested formulations, the 1:2 aronia:β-glucan system showed the most favorable behavior and may represent a useful starting point for further development. However, the findings should be interpreted as preliminary and formulation-oriented, and further studies under broader application-relevant conditions are needed to confirm the technological and biological relevance of this system. Comparison with other carrier systems would also be useful to further define the specific advantages and limitations of the aronia–β-glucan matrix.

## Figures and Tables

**Table 1:** Optimized extraction parameters for aronia fruit providing the highest predicted total polyphenol content (TPC).

Parameter	Optimal Value
Ethanol content [%]	36.9
Temperature [°C]	34.1
Ratio [mL/g]	54.5

**Table 2:** Content of major bioactive compounds identified in the optimized *Aronia melanocarpa* extract by HPLC (cyanidin-3-O-galactoside, cyanidin-3-O-arabinoside, neochlorogenic acid, and chlorogenic acid).

Active Compound	Content mg/g Lyophilized Extract
Cyanidin-3-O-galactoside	0.240 ± 0.001
Cyanidin-3-O-arabinoside	0.103 ± 0.001
Neochlorogenic acid	2.173 ± 0.041
Chlorogenic acid	3.085 ± 0.087

**Table 3:** Inhibitory activity (IC_50_ values) of the aronia extract and acarbose against α-amylase. * indicates a statistically significant difference between aronia extract and acarbose within the same assay (*p* < 0.05).

	Chokeberry Extract	Acarbose
α-amylase	3.18 ± 0.27 mg/mL *	6.76 ± 0.21 mg/mL

**Table 4:** Antioxidant activity (IC_50/0.5_ values) of the aronia extract and ascorbic acid. Values are presented as mean ± SD. * indicates a statistically significant difference between aronia extract and ascorbic acid within the same assay (*p* < 0.05).

	Chokeberry Extract	Ascorbic Acid
ABTS	1.86 ± 0.08 mg/mL *	0.089 ± 0.002 mg/mL
CUPRAC	0.43 ± 0.01 mg/mL *	0.073 ± 0.003 mg/mL

**Table 5:** First-order thermal degradation kinetics of anthocyanins in lyophilized *Aronia melanocarpa* extract and aronia–β-glucan systems at 85 °C, expressed as degradation rate constant (k), half-life (t_1/2_), and goodness of fit (R^2^). Different superscript letters within the same column indicate statistically significant differences between formulations according to Tukey’s post hoc test (*p* < 0.05).

System	k × 10^−5^ (s^−1^)	t_1/2_ (h)	R^2^
Aronia	9.56 ± 0.68 ^c^	2.019 ± 0.143 ^a^	0.979
Aronia:β-glucan System 1:1	7.54 ± 0.28 ^b^	2.644 ± 0.043 ^a^	0.982
Aronia:β-glucan System 2:1	6.70 ± 0.51 ^b^	3.900 ± 0.155 ^a^	0.996
Aronia:β-glucan System 1:2	2.05 ± 0.18 ^a^	10.672 ± 0.672 ^b^	0.997

**Table 6:** Box–Behnken experimental design matrix for the optimization of *Aronia melanocarpa* extraction parameters, including ethanol concentration (% *v*/*v*), extraction temperature (°C), and solvent-to-solid ratio (mL/g). Central points are indicated as (C).

Nr	Ethanol Content (%)	Temperature (°C)	Ratio (mL/g)
1	0	20	40
2	100	20	40
3	0	70	40
4	100	70	40
5	0	47.5	20
6	100	47.5	20
7	0	47.5	60
8	100	47.5	60
9	50	20	20
10	50	70	20
11	50	20	60
12	50	70	60
13 (C)	50	47.5	40
14 (C)	50	47.5	40
15 (C)	50	47.5	40

## Data Availability

The data supporting the findings of this study are openly available in Zenodo at DOI: 10.5281/zenodo.20127553.

## Supplementary Materials

The following supporting information can be downloaded at: https://www.mdpi.com/article/10.3390/molecules31132204/s1, Section S1: Molecular Docking and Molecular Dynamics Simulation Details; Figures S1–S4 and Tables S1–S4: minimum distance factor analyses for HPA–CGA, HPA–NCGA, HPA–C3G, and HPA–C3A systems; Figures S5–S8: RMSD analyses of selected HPA–C3G and HPA–C3A systems; Section S2: HPLC Method Validation; Table S5: HPLC validation parameters.

## Abbreviations

The following abbreviations are used in this manuscript:

ACPYPE	AnteChamber PYthon Parser interfacE
ATR	attenuated total reflectance
C3A	cyanidin-3-O-arabinoside
C3G	cyanidin-3-O-galactoside
CGA	chlorogenic acid
CP	contact probability
DNSA	3,5-dinitrosalicylic acid
FT-IR	Fourier-transform infrared spectroscopy
GAE	gallic acid equivalents
GAFF2	General Amber Force Field 2
HPA	human pancreatic α-amylase
HPLC	high-performance liquid chromatography
IC_50_	half inhibitory concentration
LINCS	Linear Constraint Solver
MD	molecular dynamics
NCGA	neochlorogenic acid
NPT	constant number of particles, pressure, and temperature
NVT	constant number of particles, volume, and temperature
PDB	Protein Data Bank
RMSD	root mean square deviation
RSM	response surface methodology
SGF	simulated gastric fluid
SIF	simulated intestinal fluid
TIP3P	transferable intermolecular potential with 3 points
TPC	total polyphenol content
VMD	Visual Molecular Dynamics
