# Supplementary material for: Antidiabetic Potential of Aronia melanocarpa–β-Glucan System: From Extraction Optimization Through In Silico Understanding of Activity to Stabilization of Anthocyanins
Source: Molecules. 2026 Jun 23;31(13):2204. doi: 10.3390/molecules31132204 (PMC13363583; doi:10.3390/molecules31132204)
Supplement: Supplementary file 1 [file molecules-31-02204-s001.zip › molecules-4344355-supplementary.pdf]

Supplementary Materials

Antidiabetic Potential of *Aronia melanocarpa*- β-Glucan System: From Extraction Optimization through In Silico Understanding of Activity to Stabilization of Anthocyanins

Anna Gościniak[1], Emmanuelle Lainé[2], Sandrine Chalancon[2], Filip Stojceski[3], Natalia Rosiak[1], Gabriele Maroni[3], Judyta Cielecka-Piontek[1]

[1] Poznan University of Medical Sciences, Department of Pharmacognosy and BiomaterialsRokietnicka 3, 60-806 Poznan, Poland; agosciniak@ump.edu.pl (A.G.); nrosiak@ump.edu.pl (N.R.)
[2] UMR 454 MEDIS UCA-INRAE, Université Clermont Auvergne, 63000 Clermont-Ferrand, France emmanuelle.laine@uca.fr (E.L); sandrine.chalancon@uca.fr (S.C.)
[3] Dalle Molle Institute for Artificial Intelligence, USI-SUPSI, Polo Universitario Lugano - Campus Est, Via La Santa, Lugano-Viganello, 6962, Switzerland filip.stojceski@supsi.ch (F.S); gabriele.maroni@supsi.ch (G.M)
* Correspondence: jpiontek@ump.edu.pl

S1. Molecular Docking and Molecular Dynamics Simulation Details

Partial atomic charges for all compounds were derived using the abcg2 charge method[1]. Chlorogenic acid and neochlorogenic acid carried a total charge of -1, while cyanidin-3-O-galactoside and cyanidin-3-O-arabinoside were modeled as neutral molecules. Ligand topologies were generated using the ACPYPE[2] tool in combination with the General Amber Force Field 2 (GAFF2)[3], following protocols previously applied in literature[4,5].

Each protein–ligand complex was placed at the center of a dodecahedral simulation box with a minimum distance of 1.2 nm between periodic images and solvated using explicit water molecules. Sodium and chloride ions were added to neutralize the systems and to reproduce physiological ionic strength (150 mM), resulting in systems comprising approximately 82,000 interacting particles. All systems were energy-minimized using the steepest descent algorithm. Subsequently, equilibration was carried out under position restraints by gradually heating the systems to 310 K (τ_t = 1 ps) over 200 ps in the NVT ensemble, followed by 200 ps of equilibration in the NPT ensemble at 1 atm (τ_p = 5 ps). Temperature and pressure were controlled using the V-rescale[6] and C-rescale[7] coupling schemes, respectively, during both equilibration and production phases.

Production MD simulations were performed in the NPT ensemble at 310 K (τ_t = 1 ps) and 1 atm (τ_p = 5 ps), with a total simulation time of 200 ns per replica. A time step of 4 fs was enabled via a hydrogen mass repartitioning scheme[8], in which the minimum atomic mass was scaled by a factor of 3, in combination with LINCS constraints[9]. The Amber ff19SB[10] force field was used to describe the protein, while the TIP3P[11]

model was employed for water molecules. All simulations were performed using GROMACS 2025.4[12,13]. Trajectory visualization and inspection were performed using Visual Molecular Dynamics (VMD) [14]. For each ligand, the 10 selected docking poses were simulated in three independent replicas. Unless otherwise stated, all analyses were performed considering the last 50 ns of each replica.

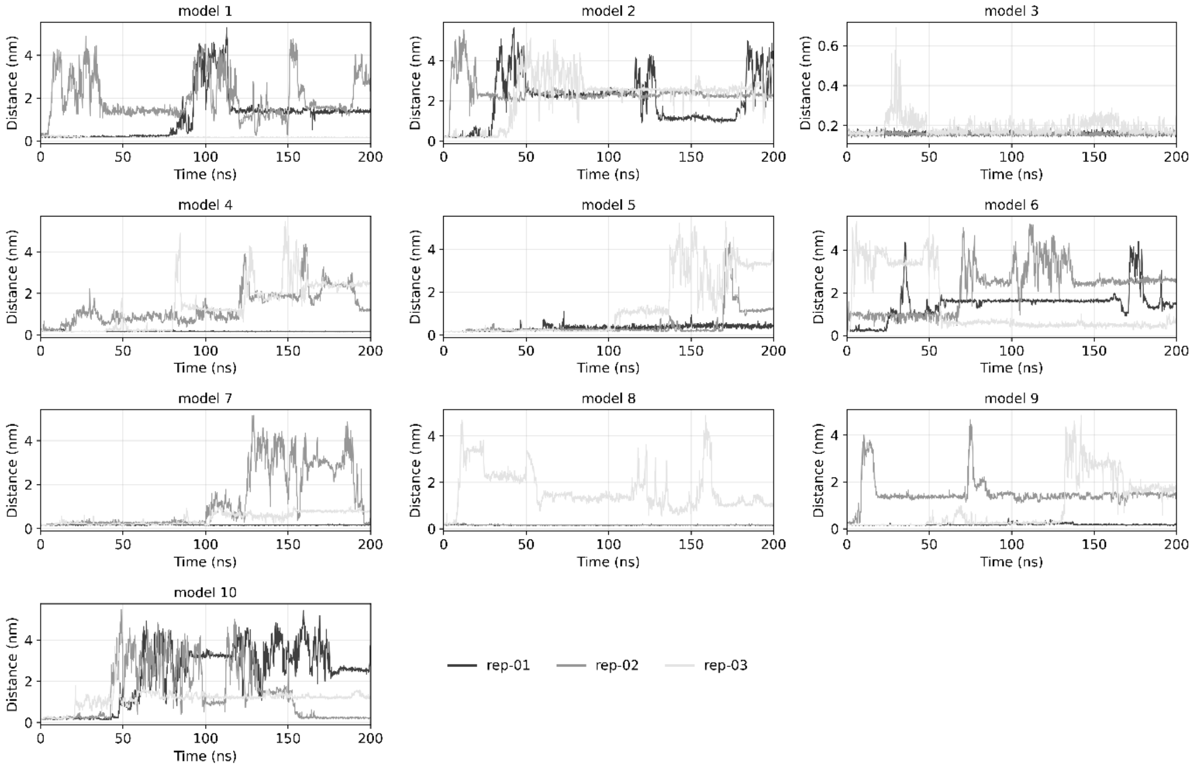

Figure S1: Minimum distance analysis between the pancreatic α-amylase (HPA) binding pocket residues and chlorogenic acid (CGA).

Table S1: Average and standard deviation of the minimum distance factor analysis between the pancreatic α-amylase (HPA) binding pocket residues and chlorogenic acid (CGA).

HPA-CGA systems	*Rep-1*	*Rep-2*	*Rep-3*
model-01	6.3521 ± 5.8951	11.3023 ± 5.6403	0.9822 ± 0.1294
model-02	11.3095 ± 6.6893	13.6093 ± 3.1985	12.8307 ± 6.4376
model-03	0.9140 ± 0.0424	0.9114 ± 0.0409	1.0901 ± 0.2745
model-04	0.9508 ± 0.1001	7.4589 ± 4.3763	7.7072 ± 6.4143
model-05	1.8731 ± 0.7068	2.5395 ± 3.2228	7.4161 ± 8.0400
model-06	8.3132 ± 3.8773	12.4611 ± 5.9999	7.7257 ± 7.9139
model-07	0.9432 ± 0.0983	7.4377 ± 7.8358	2.6461 ± 1.4823
model-08	0.9264 ± 0.0537	0.9306 ± 0.0798	9.3766 ± 4.8229
model-09	1.0180 ± 0.1929	8.5004 ± 3.1124	5.3821 ± 6.0484
model-10	13.1660 ± 8.1010	8.2560 ± 7.6592	6.5440 ± 2.3413

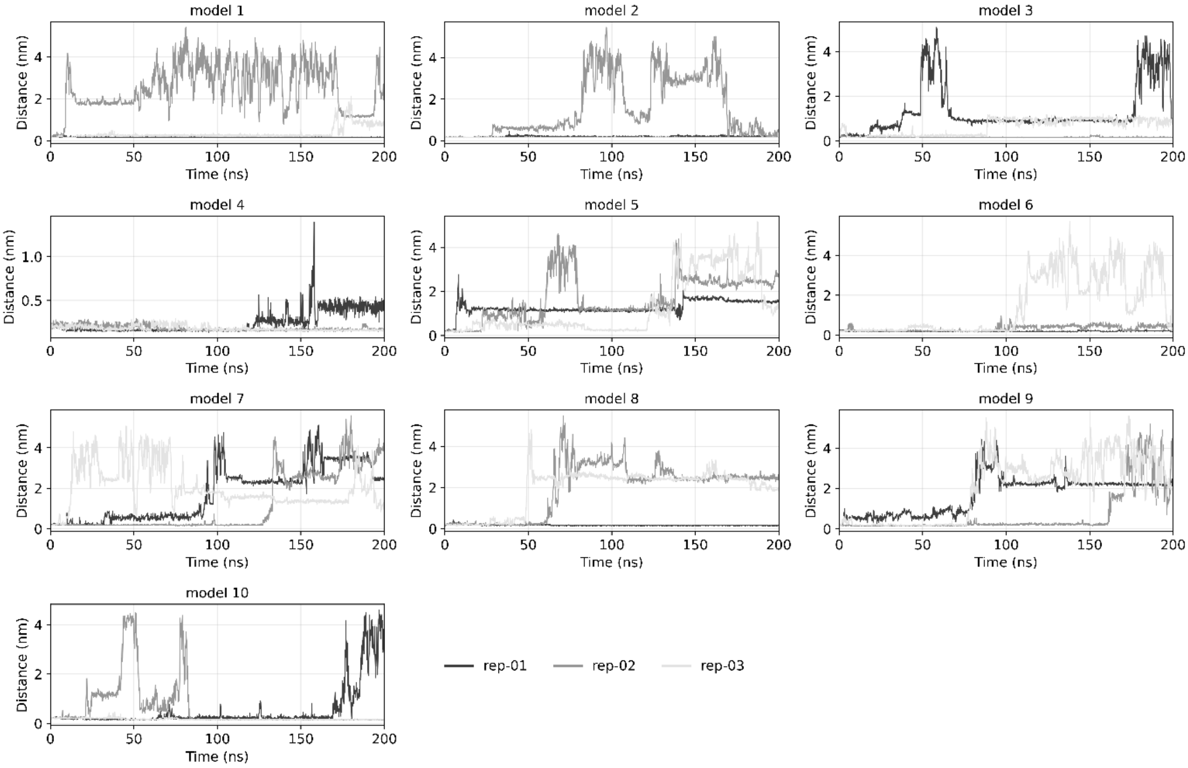

Figure S2: Minimum distance analysis between the pancreatic α-amylase (HPA) binding pocket residues and neochlorogenic acid (NCGA).

Table S2: Average and standard deviation of the minimum distance factor analysis between the pancreatic α-amylase (HPA) binding pocket residues and neochlorogenic acid (NCGA).

HPA-NCGA systems	*Rep-1*	*Rep-2*	*Rep-3*
model-01	0.9275 ± 0.0635	14.3901 ± 6.2761	1.9658 ± 1.5529
model-02	1.1256 ± 0.1722	8.9199 ± 8.0487	0.9151 ± 0.0490
model-03	7.4601 ± 6.1654	0.9457 ± 0.0987	3.5746 ± 2.0357
model-04	1.3983 ± 0.7211	1.0454 ± 0.1740	1.0311 ± 0.1631
model-05	7.2190 ± 1.7023	9.4037 ± 5.5690	7.2950 ± 7.5023
model-06	1.0719 ± 0.1464	1.8262 ± 0.7772	8.3851 ± 8.4805
model-07	9.9945 ± 7.4415	6.6709 ± 7.9919	11.6176 ± 5.8257
model-08	0.9754 ± 0.1473	11.0900 ± 7.1295	11.0487 ± 5.5946
model-09	9.5621 ± 5.0552	3.9012 ± 6.3728	11.3856 ± 8.8767
model-10	3.0277 ± 4.9974	3.5469 ± 5.4386	1.0150 ± 0.1865

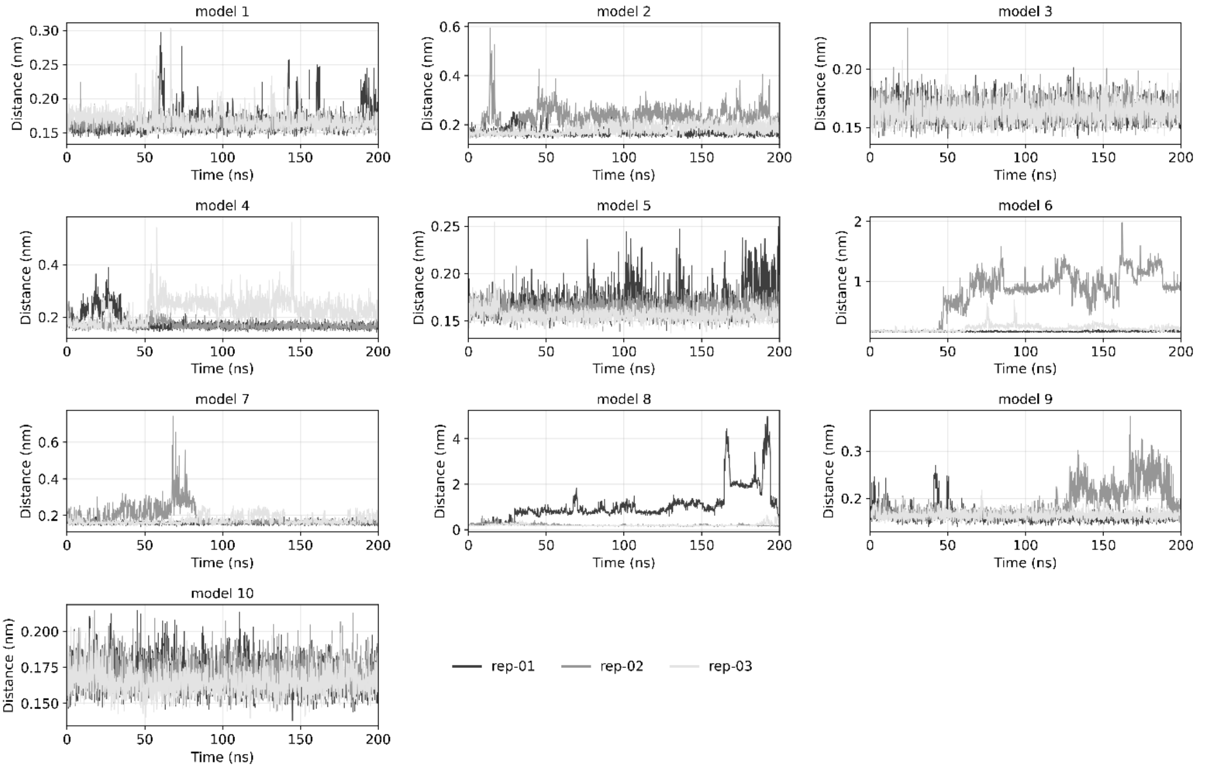

Figure S3: Minimum distance analysis between the pancreatic α-amylase (HPA) binding pocket residues and cyanidin-3-O-galactoside (C3G).

Table S3: Average and standard deviation of the minimum distance factor analysis between the pancreatic α-amylase (HPA) binding pocket residues and cyanidin-3-O-galactoside (C3G).

HPA-C3G systems	*Rep-1*	*Rep-2*	*Rep-3*
model-01	0.9452 ± 0.1022	0.9334 ± 0.0445	0.9573 ± 0.0743
model-02	0.9605 ± 0.0919	1.3090 ± 0.2470	1.0192 ± 0.1159
model-03	0.9374 ± 0.0503	0.9411 ± 0.0536	0.9389 ± 0.0521
model-04	1.0069 ± 0.1651	0.9680 ± 0.0761	1.2888 ± 0.2562
model-05	0.9629 ± 0.0915	0.9314 ± 0.0461	0.8954 ± 0.0400
model-06	0.9263 ± 0.0483	4.4351 ± 2.2133	1.2143 ± 0.2962
model-07	0.9085 ± 0.0428	1.0771 ± 0.2965	0.9879 ± 0.1294
model-08	5.9979 ± 4.2004	1.1417 ± 0.2790	1.1126 ± 0.3067
model-09	0.9256 ± 0.0792	1.0698 ± 0.1977	0.9420 ± 0.0491
model-10	0.9614 ± 0.0627	0.9679 ± 0.0587	0.9438 ± 0.0529

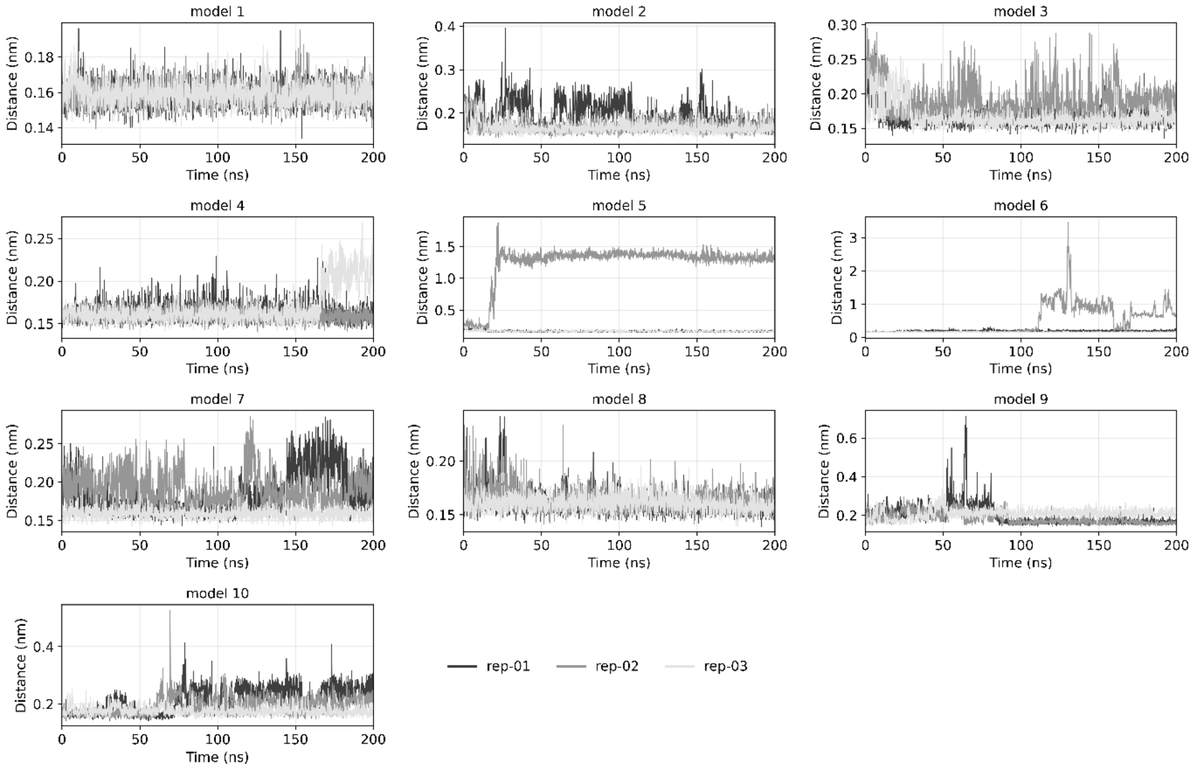

Figure S4: Minimum distance analysis between the pancreatic α-amylase (HPA) binding pocket residues and cyanidin-3-O-arabinoside (C3A).

Table S4: Average and standard deviation of the minimum distance factor analysis between the pancreatic α-amylase (HPA) binding pocket residues and cyanidin-3-O- arabinoside (C3A).

HPA-C3A systems	*Rep-1*	*Rep-2*	*Rep-3*
model-01	0.9063 ± 0.0375	0.9064 ± 0.0364	0.9099 ± 0.0376
model-02	1.1143 ± 0.1764	0.9718 ± 0.0888	0.9528 ± 0.0779
model-03	0.9402 ± 0.0756	1.0817 ± 0.1462	0.9553 ± 0.0962
model-04	0.9402 ± 0.0599	0.9160 ± 0.0425	0.9663 ± 0.1174
model-05	0.9515 ± 0.0750	7.1039 ± 1.8099	0.9485 ± 0.0697
model-06	1.0959 ± 0.1364	2.7757 ± 2.4895	0.9256 ± 0.0492
model-07	1.0493 ± 0.1559	1.0886 ± 0.1219	0.9100 ± 0.0379
model-08	0.9258 ± 0.0623	0.9387 ± 0.0616	0.9188 ± 0.0433
model-09	1.1313 ± 0.3056	1.0521 ± 0.1684	1.1957 ± 0.1354
model-10	1.2140 ± 0.2522	1.0991 ± 0.1783	1.0074 ± 0.0901

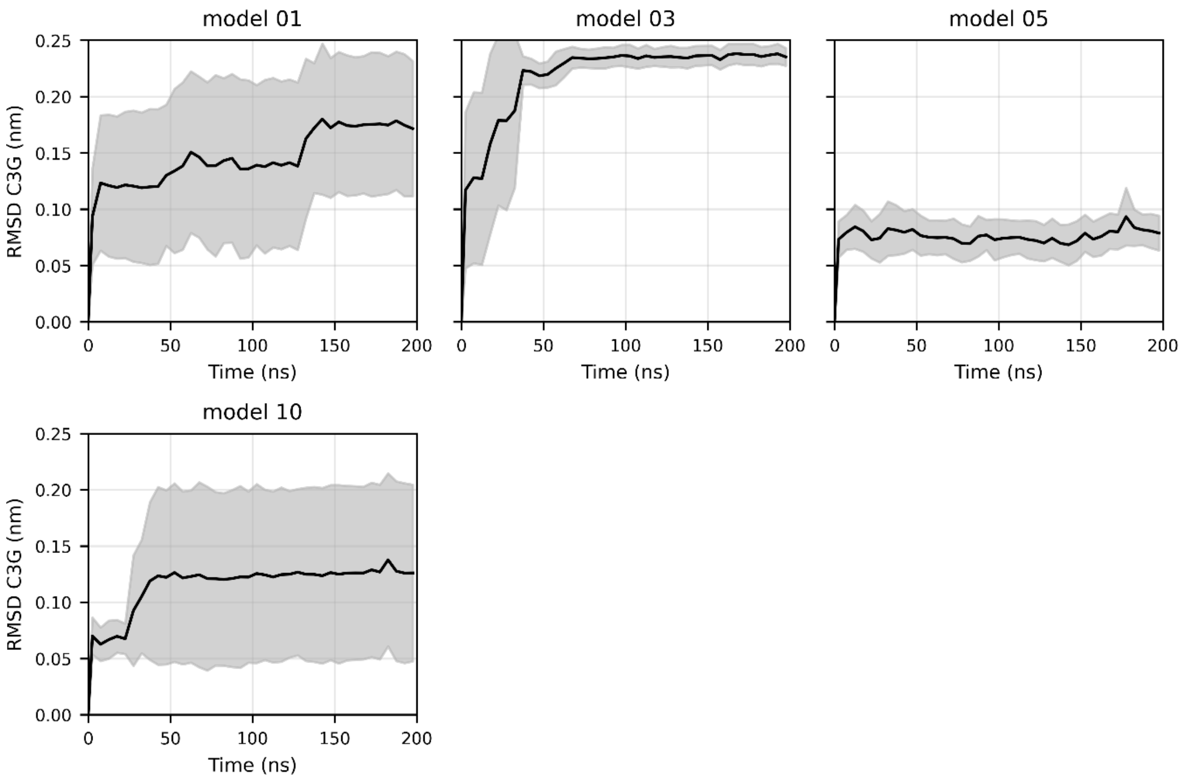

Figure S5: Root-mean-square deviation analysis (RMSD) of the cyanidin-3-O-galactoside (C3G) ligand that have minimum distance factor <1 in all 3 replicas. The RMSD analyses are presented as the average (black line) with the corresponding standard deviation (grey shaded area), calculated using 5 ns time windows.

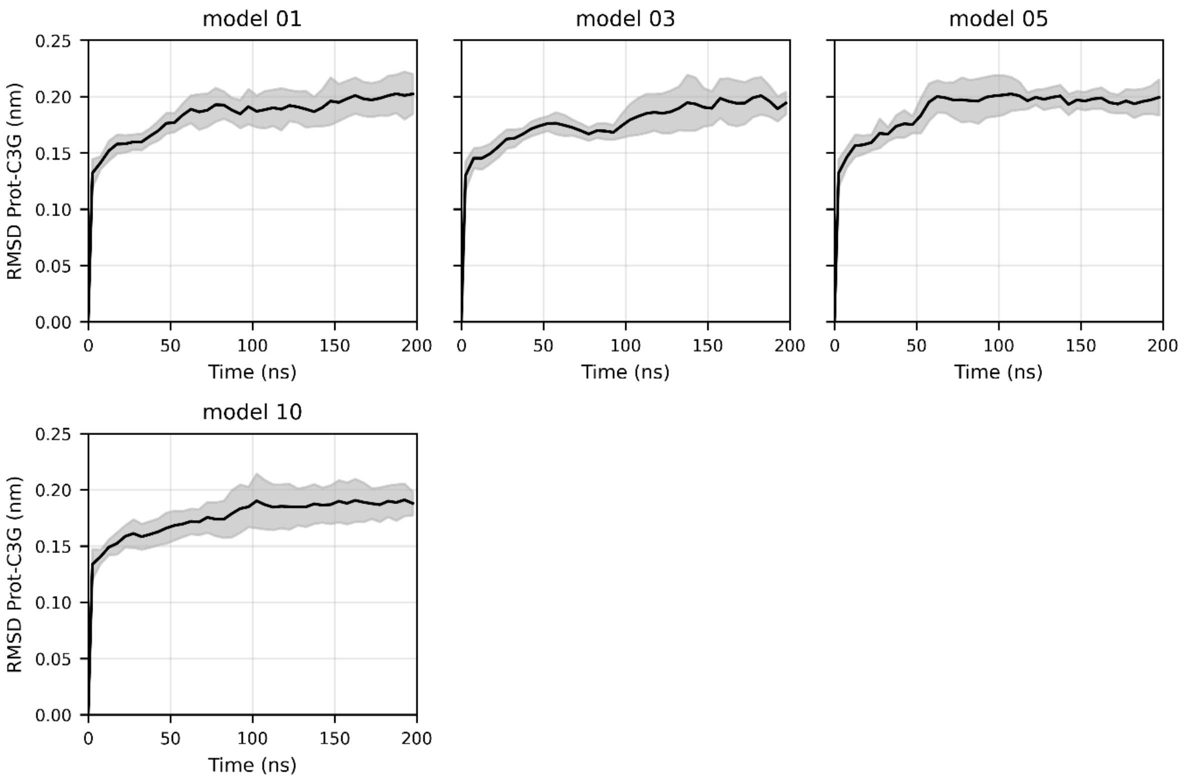

Figure S6: Root-mean-square deviation analysis (RMSD) of the protein-ligand complex (HPA-C3G) that have minimum distance factor <1 in all 3 replicas. The RMSD analyses are presented as the average (black line) with the corresponding standard deviation (grey shaded area), calculated using 5 ns time windows.

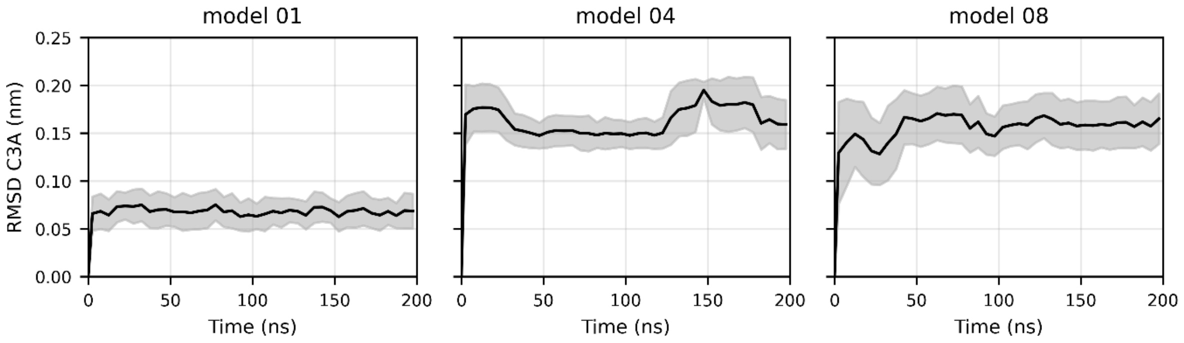

Figure S7: Root-mean-square deviation analysis (RMSD) of the cyanidin-3-O-arabinoside (C3A) ligand that have minimum distance factor <1 in all 3 replicas. The RMSD analyses are presented as the average (black line) with the corresponding standard deviation (grey shaded area), calculated using 5 ns time windows.

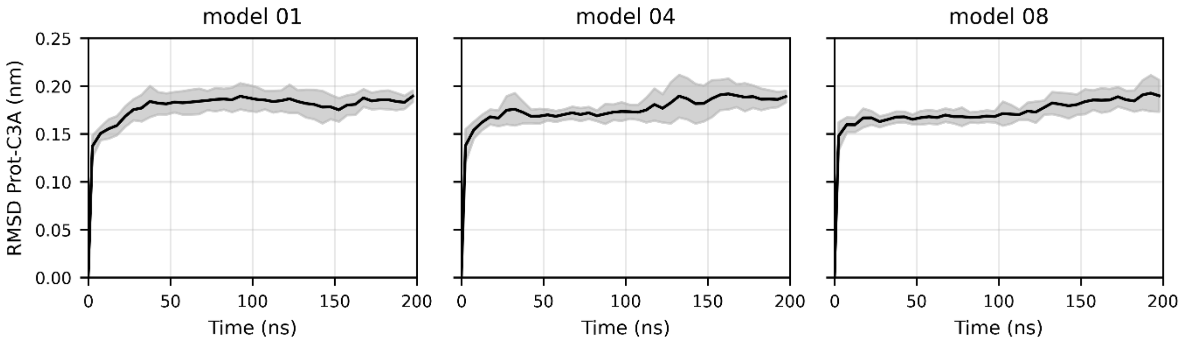

Figure S8: Root-mean-square deviation analysis (RMSD) of the protein-ligand complex (HPA-C3A) that have minimum distance factor <1 in all 3 replicas. The RMSD analyses are presented as the average (black line) with the corresponding standard deviation (grey shaded area), calculated using 5 ns time windows.

S2. HPLC Method Validation

Table S5: Validation parameters of the HPLC method used for the determination of cyanidin glycosides and phenolic acids in Aronia melanocarpa extract.

**Validation parameter**	**Cyanidin-3-O-galactoside**	**Cyanidin-3-O-arabinoside**	**Chlorogenic acid**	**Neochlorogenic acid**
**Linearity: y = ax + b**				
$a \pm S_a$	43791.949	39976.023	27641.347	24815.437
$b \pm S_\beta$	n.s.	n.s.	n.s.	n.s.
Correlation coefficient (r)	0.999834	0.999837	0.999752	0.999749
Range of linearity [µg/mL]	0.24–2.38	0.54–5.410	5.92–59.20	4.20–42.00
Limit of detection (LOD) [µg/mL]	0.032	0.072	1.201	0.857
Limit of quantification (LOQ) [µg/mL]	0.097	0.218	3.639	2.597

1. He, X.; Man, V.H.; Yang, W.; Lee, T.-S.; Wang, J. ABCG2: A Milestone Charge Model for Accurate Solvation Free Energy Calculation. *J. Chem. Theory Comput.* **2025**, *21*, 3032–3043, doi:10.1021/acs.jctc.5c00038.

2. Sousa Da Silva, A.W.; Vranken, W.F. ACPYPE - AnteChamber PYthon Parser interfacE. *BMC Res Notes* **2012**, *5*, 367, doi:10.1186/1756-0500-5-367.

3. Wang, J.; Wolf, R.M.; Caldwell, J.W.; Kollman, P.A.; Case, D.A. Development and Testing of a General Amber Force Field. *J. Comput. Chem.* **2004**, *25*, 1157–1174, doi:10.1002/jcc.20035.

4. Stojceski, F.; Grasso, G.; Pallante, L.; Danani, A. Molecular and Coarse-Grained Modeling to Characterize and Optimize Dendrimer-Based Nanocarriers for Short Interfering RNA Delivery. *ACS Omega* **2020**, *5*, 2978–2986, doi:10.1021/acsomega.9b03908.

5. Stojceski, F.; Buetti-Dinh, A.; Stoddart, M.J.; Danani, A.; Della Bella, E.; Grasso, G. Influence of Dexamethasone on the Interaction between Glucocorticoid Receptor and SOX9: A Molecular Dynamics Study. *Journal of Molecular Graphics and Modelling* **2023**, *125*, 108587, doi:10.1016/j.jmgm.2023.108587.

6. Bussi, G.; Donadio, D.; Parrinello, M. Canonical Sampling through Velocity Rescaling. *The Journal of Chemical Physics* **2007**, *126*, 014101, doi:10.1063/1.2408420.

7. Bernetti, M.; Bussi, G. Pressure Control Using Stochastic Cell Rescaling. *The Journal of Chemical Physics* **2020**, *153*, 114107, doi:10.1063/5.0020514.

8. Hopkins, C.W.; Le Grand, S.; Walker, R.C.; Roitberg, A.E. Long-Time-Step Molecular Dynamics through Hydrogen Mass Repartitioning. *J. Chem. Theory Comput.* **2015**, *11*, 1864–1874, doi:10.1021/ct5010406.

9. Hess, B.; Bekker, H.; Berendsen, H.J.C.; Fraaije, J.G.E.M. LINCS: A Linear Constraint Solver for Molecular Simulations. *J. Comput. Chem.* **1997**, *18*, 1463–1472, doi:10.1002/(SICI)1096-987X(199709)18:12<1463::AID-JCC4>3.0.CO;2-H.

10. Tian, C.; Kasavajhala, K.; Belfon, K.A.A.; Raguette, L.; Huang, H.; Migues, A.N.; Bickel, J.; Wang, Y.; Pincay, J.; Wu, Q.; et al. ff19SB: Amino-Acid-Specific Protein Backbone Parameters Trained against Quantum Mechanics Energy Surfaces in Solution. *J. Chem. Theory Comput.* **2020**, *16*, 528–552, doi:10.1021/acs.jctc.9b00591.

11. Jorgensen, W.L.; Chandrasekhar, J.; Madura, J.D.; Impey, R.W.; Klein, M.L. Comparison of Simple Potential Functions for Simulating Liquid Water. *The Journal of Chemical Physics* **1983**, *79*, 926–935, doi:10.1063/1.445869.

12. Kutzner, C.; Páll, S.; Fechner, M.; Esztermann, A.; Groot, B.L.; Grubmüller, H. More Bang for Your Buck: Improved Use of GPU Nodes for GROMACS 2018. *J Comput Chem* **2019**, *40*, 2418–2431, doi:10.1002/jcc.26011.

13. Abraham, M.J.; Murtola, T.; Schulz, R.; Páll, S.; Smith, J.C.; Hess, B.; Lindahl, E. GROMACS: High Performance Molecular Simulations through Multi-Level Parallelism from Laptops to Supercomputers. *SoftwareX* **2015**, *1–2*, 19–25, doi:10.1016/j.softx.2015.06.001.

14. Humphrey, W.; Dalke, A.; Schulten, K. VMD: Visual Molecular Dynamics. *Journal of Molecular Graphics* **1996**, *14*, 33–38, doi:10.1016/0263-7855(96)00018-5.
